# Maternal investment, maturational rate of the offspring and mechanical competence of the adult female skeleton

**DOI:** 10.1093/emph/eoy015

**Published:** 2018-08-16

**Authors:** Alison A Macintosh, Jonathan C K Wells, Jay T Stock

**Affiliations:** 1 Department of Archaeology, University of Cambridge, Cambridge, UK; 2 Childhood Nutrition Research Centre, UCL Great Ormond Street Institute of Child Health, London WC, UK; 3 Department of Anthropology, Western University, London, ON, Canada

**Keywords:** life history, fracture risk, women's health, development

## Abstract

**Lay summary:**

Girls with a slower life history trajectory build a larger body with larger and mechanically stronger bones. Thus, variation in the emergence of slower versus faster life history trajectories during development can have consequences for bone mechanical competence, and hence fracture risk in adulthood.

**Background and objectives:**

Variation in life history trajectory, specifically relative investment in growth versus reproduction, has been associated with chronic disease risk among women, but whether this scenario extends to skeletal health and fracture risk is unknown. This study investigates the association of life history traits (proxies for maternal investment and maturational rate) with female bone outcomes in adulthood.

**Methodology:**

Body size variables, regional muscle and fat areas, and cross-sectional bone size and strength outcomes were obtained from 107 pre-menopausal women encompassing a wide range of physical activity levels. Developmental parameters (birth weight, age at menarche) were obtained from questionnaires.

**Results:**

High birth weight was significantly associated with a proportionately larger body and larger, mechanically stronger bones, independently of physical activity level. It was also positively but non-significantly associated with age at menarche. Later menarche was significantly associated with larger and mechanically stronger bones and substantially less absolute and relative regional subcutaneous fat. Age at menarche exhibited stronger relationships with adult adiposity than did physical activity.

**Conclusions and implications:**

Both larger birth weight and later menarche contribute to a slower life history trajectory, which is associated with greater body size, leanness and bone mechanical competence in early adulthood. In contrast, earlier sexual maturity prioritized energy allocation in adiposity over body size and skeletal strength. Thus, the level of maternal investment and the woman’s own life history trajectory shape investment in skeletal properties, with implications for fracture risk later in life.

## INTRODUCTION

There is now compelling evidence that patterns of nutrition and growth in early life predict diverse components of health in later life. On the one hand, birth weight is inversely associated with the risk of chronic non-communicable disease (NCD) in adulthood [1], while on the other, rapid weight gain in post-natal life increases risk of the same diseases [2]. These findings have given rise to the ‘developmental origins of adult health and disease hypothesis’ [3]. In adulthood, both short stature and elevated weight and adiposity are associated with greater NCD risk [4–7], highlighting how developmental trajectory shapes adult health in the long term.

These data can be reinterpreted using a life-history perspective [8, 9]. According to life history theory, metabolic resources at any stage of the life cycle must be allocated across four competing functions: maintenance, growth, reproduction and defence against pathogens and predators [10, 11]. Greater allocation of energy to one of these functions results in less energy being available for the others. When the theory was first developed, particular attention was paid to the impact of these ‘allocation decisions’ on extrinsic mortality risk. The lower the likelihood of long-term survival, the greater the fitness payoffs of diverting energy towards immediate survival and imminent reproduction, thus constraining investment in maintenance [12]. As extrinsic mortality risk declines, the returns on investment in maintenance increase: extending the duration of the reproductive career allows more offspring to be produced, ultimately increasing fitness.

More recently, the same explanatory framework has been applied to ‘intrinsic’ components of mortality risk and longevity [13, 14]. Given that early growth patterns have long-term implications for the rate of ageing and the ability to buffer infectious diseases, poor early growth is indicative of a shorter lifespan. Conversely, greater growth during foetal life indicates higher intrinsic quality of the body, predictive of a longer lifespan. Fitness can then be maximised by investing more in growth and maintenance through a lengthier period of development, ultimately prolonging the duration of the reproductive career and increasing the capacity to invest in offspring.

On this basis, the pace of life history is predicted to be an important trait shaping the profile of health and disease in adult life. Both the magnitude of nutritional investment during early critical windows and the pace of maturation are predicted to shape adult NCD risk. Indeed, these two traits might be linked, since a faster pace of life history may develop if the quality of early development was poor. Supporting this hypothesis, lower investment in foetal life (proxied by birth weight) was associated with faster maturation, short adult stature, higher levels of body fat and high blood pressure in South Asian women living in the UK [13]. These data indicate that in an environment where energy supply in postnatal life was not constrained, those who had achieved poorer foetal development adopted faster life history strategies, prioritizing reproduction over growth, maintenance and health.

More specifically, the pace of life history should explain differential investment across a range of phenotypic outcomes that orchestrate trade-offs across both somatic traits and life history functions. In a related review article, we argue that the pace of life history of mothers is associated with their body size, composition and metabolic profile, their NCD risk, and their capacity to invest in the next generation [9]. Faster female life histories are associated with shorter height, smaller pelvises and lower lean mass, all of which constrain mothers’ investment in their offspring during pregnancy. If ecological conditions permit, they resolve this by investing more in their offspring during lactation, and benefit from elevated adiposity. However, this pattern prevents the offspring acquiring energy during the critical window most influential for long-term health, namely foetal life [9].

The pace of life history is therefore expected to shape not only body size and composition, but also other components of health associated with the life history function categorized as ‘maintenance’ [9]. Here, we test this hypothesis in relation to female bone health and mechanical competence in adult life, whilst controlling broadly for variation in physical activity. The mechanical competence of bone is determined by a combination of bone quality (e.g. material properties, microstructure, density) and bone architecture (e.g. cross-sectional moments of inertia like *I*_max_) [15]. Bone quality is largely genetically determined [16], but bone architecture demonstrates substantial plasticity during life, particularly in response to mechanical loading [17–22]. As a result, when bone quality and architecture are considered together, for example when using indices such as the Bone Strength Index (BSI) or strength-strain index (SSI), it is the architectural component that most contributes to variation in mechanical competence [15]. The spatial distribution of bone tissue plays a major role in its bending and torsional rigidity during loading [23], and increased cross-sectional size may play a major role in reducing fracture risk, even among bones of comparable tissue quality [16]. Thus, in this study, we consider bone outcomes that reflect bone size, spatial distribution and strength to be indicative of investment in ‘maintenance’ from a life history perspective, as well as bone quality (total bone mineral density). We evaluate the relationships between these parameters, soft tissue areas and markers of maternal investment and maturational rate, in order to determine the extent to which the pace of life history interacts with physical activity in contributing to skeletal integrity among pre-menopausal women. These relationships may provide valuable insight into the importance of maternal health and developmental pace in shaping daughters’ bone strength and long-term fracture risk.

## METHODOLOGY

### Subjects

All participants were healthy adults, predominantly of European descent living in the United Kingdom, and all were between the ages of 18 and 40 years. The following exclusion criteria were established prior to recruitment and were applied to all subjects: any medical condition or medication known to interfere with bone metabolism, any current or recent (past 12 months) pregnancy or lactation, 18 years of age or younger (for bone scanning only), or post-menopausal status. Participants were divided into two groups according to their current physical activity level, either ‘recreationally active’ or ‘competitive athlete.’ Recreationally active women were those that had never participated in competitive sport, and had no current or past participation in >3 h a week of weight-bearing intensive physical activity. Competitive athletes were those who had been training and competing intensively in rowing, football (soccer), or endurance running for at least the past 3 years. All participants were recruited through the Cambridge University Women’s Boat Club, Women’s Association Football Club, Athletics Club, Hare and Hounds and Triathlon Club, as well as the Cambridge and Coleridge Athletics Club, the Cambridge Triathlon Club, the Beyond the Ultimate Jungle Ultra 2016, the Everest Trail Race 2016, several University of Cambridge colleges and the Graduate Union. Participants were recruited as part of two separate studies approved by the Cambridge University Human Biology Research Ethics Board (HBREC.2015.25 and HBREC.2016.14). Ethical approval for the use of peripheral quantitative computed tomography (pQCT) was obtained from the NHS Health Research Authority NRES Committee East of England—Cambridge East (15/EE/0017). All participants provided written informed consent prior to participation, and filled out a health/activity questionnaire in which developmental parameters such as birth weight and age at menarche were obtained. Data from a total of 111 women were included in the current study (38 classified as recreationally active women and 73 as competitive athletes), 107 of whom participated in pQCT scanning.

### Anthropometry

Stature was measured to the nearest 0.1 cm using a SECA 274 stadiometer. Body mass was recorded to the nearest 0.1 kg with a SECA electronic scale. Maximum humeral, femoral and tibial lengths and bi-iliac breadths were obtained from participants using sliding calipers according to the methods in International Standards for Anthropometric Assessment [24]. Humeral length was measured as the distance between the most inferior border of the acromion and the distal border of the capitulum. Femoral length was measured as the distance between the proximal border of the greater trochanter and the distal border of the lateral condyle. Tibial length was measured as the distance between the proximal medial border of the tibial plateau and distal border of the medial condyle. Bi-iliac breadth (BIB) was measured as the distance between the left and right anterior superior iliac spines. Relative bi-iliac breadth (RBB) was quantified by dividing bi-iliac breadth by stature, to provide a measure of the relative breadth of the pelvis.

### Peripheral quantitative computed tomography

All cross-sectional bone and soft tissue data were collected using peripheral quantitative computed tomography (XCT-3000; Stratec Medizintechnik GmbH, Pforzheim, Germany) at the PAVE Imaging and Performance Lab in the Department of Archaeology at the University of Cambridge. Cross-sectional images were obtained from midshaft section locations (50% of maximum length) of the left and right humeri, right femur and right tibia, and distal epiphyseal section locations of the right femur and right tibia (4% of maximum length from the distal end). The left lower limb bone was scanned instead of the right in the case of previous injuries that may have affected bone or soft tissue morphology. Any scan in which movement artefacts were present affecting the bone and/or soft tissue was removed from analyses.

Quantification of the majority of bone and soft tissue variables was performed with Macro analyses in the manufacturer software (XCT, version 6.2.0). Cross-sectional bone size was assessed through the quantification of total areas (ToA; mm^2^) at midshaft (50%) and distal (4%) section locations. ToA provides a measure of the total cross-sectional area of the bone and marrow space. Midshaft ToA was assessed using a threshold of 710 mg/cm^3^ (contour mode 1) to separate the periosteal contour of the cortical bone from the surrounding soft tissue. Distal ToA was assessed using contour mode 1, peel mode 2, with an inner threshold of 280 mg/cm^3^ (tibia) or 220 mg/cm^3^ (femur) to separate bone from marrow, and an outer threshold of 540 mg/cm^3^ to separate cortical bone from muscle. Bone quality was assessed using femoral and tibial total bone density (TBD; mg/cm^3^) from the distal epiphysis (4% section location); this was quantified using the same thresholds as above. TBD provides a measure of the mean density of the total bone, including the cortical bone shell and trabecular bone.

Soft tissue composition was assessed through the quantification of cross-sectional total muscle and total fat areas from the midshaft location of the humerus and femur and the 66% section location of the tibia. Total limb area at these section locations was first assessed using a threshold of −52 mg/cm^3^ (contour mode 3, peel mode 1, F03F05F05 filter). Total muscle area (mm^2^) was quantified using a threshold of 41 mg/cm^3^ (contour mode 3, peel mode 1, F03F05F05 filter) to peel off the subcutaneous fat, from which the software automatically subtracted ToA. Total fat area (mm^2^) was automatically calculated as the difference between total limb area and combined total muscle and bone areas.

Bone strength was assessed through the quantification of the polar strength-strain index (SSI_p_; mm^4^) and the maximum second moment of area, or moment of inertia (*I*_max_; mm^4^). SSI_p_ provides a measure of the torsional mechanical strength of the whole bone in cross-section, based on its geometrical and material properties [25], both equally important in contributing to mechanical strength [26]. SSI_p_ was quantified from all midshaft limb sites using a threshold of 710 mg/cm^3^ (contour mode 4) to separate soft tissue from cortical bone and 400 mg/cm^3^ to separate cortical bone from marrow. Cross-sectional images of midshaft humeri, femora and tibiae were then imported into ImageJ, and the ‘Optimise Threshold’ function was used to remove soft tissue and marrow. The maximum second moment of area, or moment of inertia (*I*_max_; mm^4^), was quantified from these thresholded images using BoneJ, a bone image analysis plug-in [27]. *I*_max_ is the second moment of area about the major axis of the cross-section, and reflects maximum bending rigidity of the bone shaft [23], reflecting bone architecture but not material properties.

### Conceptual approach

Based on our related review article [9], we hypothesized that a daughter’s life history trajectory would be influenced by maternal investment during her foetal life, with less investment being associated with smaller adult size, accelerated maturation and greater fat accumulation in the offspring. As foetal growth constraint and subsequently faster life history trajectories have been associated with reduced investment in long-term ‘maintenance’ and increased adult NCD risk [13], we also expected that these developmental parameters would be associated with less investment in skeletal size and the mechanically strong spatial distribution of bone. If so, these relationships between developmental parameters and adult bone may provide insight into the importance of maternal health in daughters’ skeletal strength and future fracture risk. We used birth weight as a marker of maternal investment in foetal life; however we were unable to include gestational age as a proxy for maternal nutritional investment in this study, as it was not among the data obtained from participants in the original studies from which we acquired all data for the current analysis. Age at menarche was used a marker of the pace of maturation, and outcomes of interest were indices of body size, bone size/strength, muscularity and adiposity.

### Statistical analyses

All data distributions were checked for normality using the Kolmogorov-Smirnov test, histograms and assessments of skewness and standard error. Data were not size-standardized prior to analyses, as the contribution of life history parameters to shaping the absolute size and strength of the tibia was of interest. Non-normal data were natural log transformed prior to analyses. Independent samples *t*-tests were used to determine whether or not physical activity groups differed in age or in the developmental predictors (birth weight, age at menarche) being examined. Partial Pearson’s correlations controlling for physical activity were used to assess the relationships between developmental predictors and each of their relationships with body size, body composition and bone variables. Multiple regression analyses were used to test for the extent to which developmental parameters reflecting maternal investment (birth weight) and maturational rate (age at menarche), as well as physical activity (competitive athlete vs. recreationally active), predicted adult body size, body composition and skeletal variables. The relative distribution of fat and muscle within section slices was assessed using standardized residuals from linear regression of mean total fat area relative to mean total muscle area. All statistical analyses were conducted in SPSS version 23.

## RESULTS

**Figure 1.**
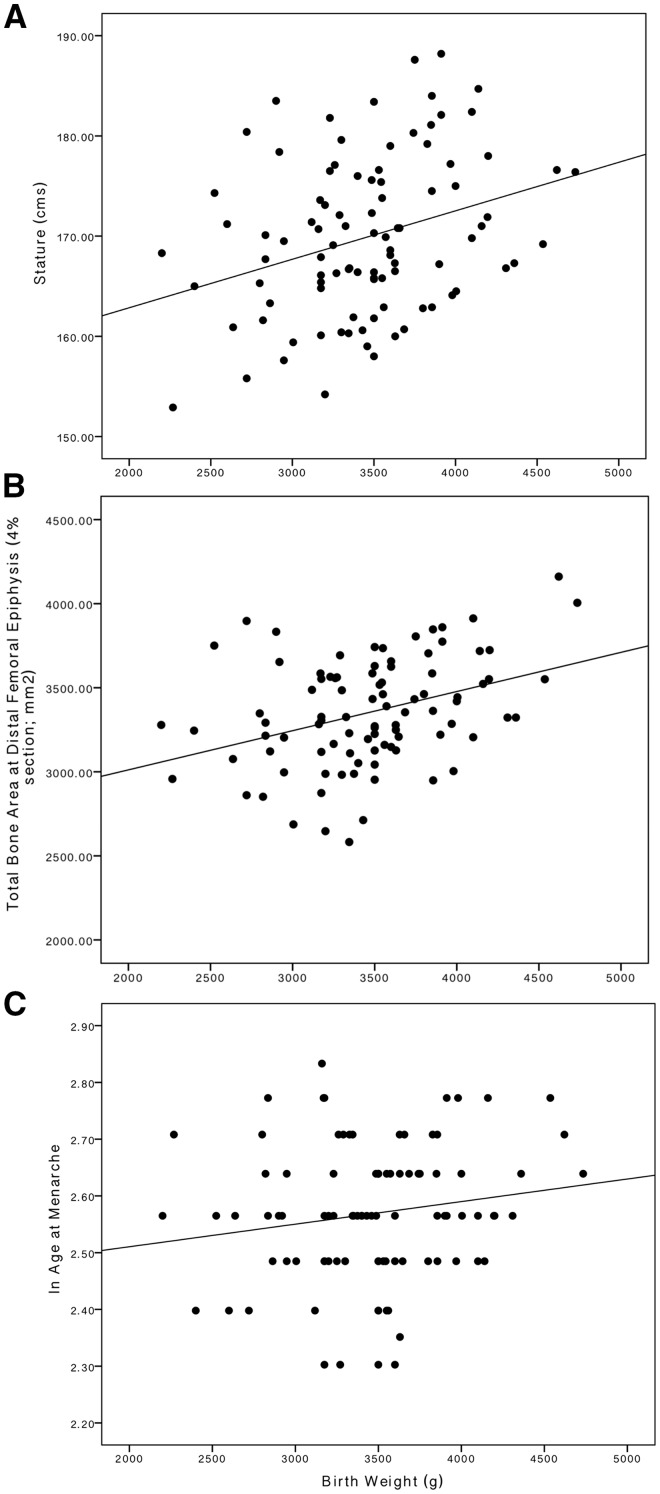
Associations between birth weight, age at menarche, adult phenotype and bone outcomes. Larger birth weight is significantly associated with: (**A**) a larger body in adulthood, (**B**) larger epiphyses in cross-section, (**C**) slightly, but not significantly, later age at menarche

**Figure 2.**
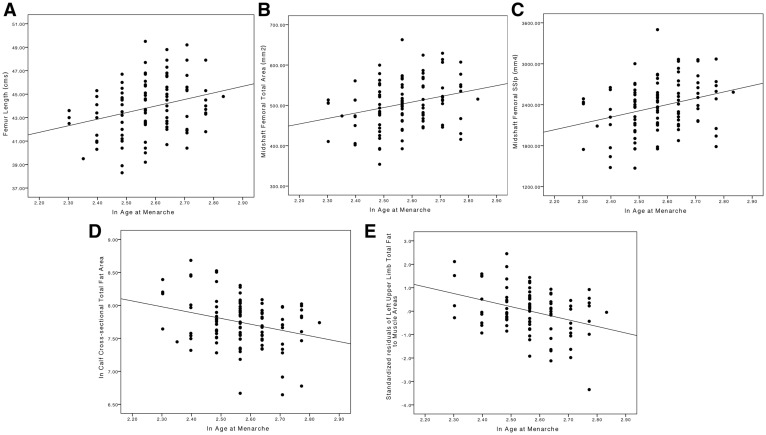
Associations between age at menarche, adult phenotype and bone outcomes. Age at menarche is significantly associated with: (**A**) longer bones in adulthood, (**B**) larger bones in cross-section at the shaft and epiphysis, (**C**) higher bending/torsional rigidity at midshaft limb bone sites, (**D**) less absolute regional subcutaneous fat in limb cross-sections and (**E**) less fat for a given amount of muscle

Summary descriptive statistics for age and developmental predictors among the full 111 participants (107 were included in bone analyses) are presented in Table 1. There were no significant differences between competitive athletes and recreationally active controls in age, birth weight or age at menarche. Results of partial Pearson’s correlations between developmental parameters (birth weight, age at menarche) and soft tissue and bone outcomes when controlling for behavioural variation are presented in Table 2. Scatterplots depicting relationships between developmental parameters and soft tissue and bone outcomes are presented in Figs 1 and 2. Birth weight was significantly associated with variables reflecting proportionately larger body size (stature, body mass, bi-iliac breadth and all bone lengths), correspondingly larger lower limb cross-sectional bone areas in the epiphyses (distal femoral and tibial ToA) and at midshaft (femoral and tibial ToA), and higher midshaft bending rigidity (left humeral and femoral *I*_max_). Birth weight was not significantly associated with any muscle or fat variables or TBD. The strongest relationships between birth weight and bone outcomes were documented in the weight-bearing lower limb, particularly the femur, with few significant relationships documented in the non-weight-bearing upper limb.

**Table 1.** Sample descriptive statistics for developmental predictors (*N* = 111:73 competitive athletes, 38 recreationally active controls)

	Mean	Standard Deviation	Range
Age (years)			
Pooled	23.5	4.2	18–40
Recreationally active	23.6	3.8	19–32
Competitive athletes	23.4	4.4	18–40
Birth weight (g)			
Pooled	3457	499	2200–4734
Recreationally active	3446	375	2720–4196
Competitive athletes	3462	556	2200–4734
Age at menarche (years)			
Pooled	13.1	1.5	10–17
Recreationally active	13.0	1.8	10–17
Competitive athletes	13.1	1.4	10–16

**Table 2.** Statistically significant partial Pearson’s correlations between developmental parameters (birth weight and age at menarche) and soft tissue and bone outcomes when controlling for athletic participation

Property	Birth weight	ln Age at menarche
Body size and shape		
Stature	0.358**	-
Body mass	0.258*	-
Bi-iliac breadth	0.284**	-
ln RBB	-	−0.253*
Left humerus length	0.243*	-
Right humerus length	0.225*	-
Femur length	0.257*	0.312**
Tibia length	0.283**	0.207*
Body Composition		
ln Total Muscle Area	-	-
ln Total Fat Area		
Left Arm	-	−0.409**
Right Arm	-	−0.420**
Thigh	-	−0.433**
Calf	-	−0.417**
ln Fat:Muscle Area		
Left Arm	-	−0.359**
Right Arm	-	−0.374**
Thigh	-	−0.410**
Calf	-	−0.353**
*Bone Size*		
ToA: Epiphysis (4%)		
Femur	0.415**	-
Tibia	0.401**	-
ToA: Midshaft		
Left humerus	-	-
Right humerus	-	0.218*
Femur	0.276*	0.247*
Tibia	0.222*	-
*Bone Strength*		
ln SSI_p_: Midshaft		
Left humerus	-	-
Right humerus	-	-
Femur	-	0.226*
ln *I*_max_: Midshaft		
Left humerus	0.220*	-
Right humerus	-	0.254*
Femur	0.268*	0.254*
*Bone Density*		
TBD: Epiphysis (4%)	-	-

* Indicates significance at *P* < 0.05;

** indicates significance at *P* < 0.01; ‘-’ indicates non-significance, *P* > 0.05.

Birth weight and age at menarche were positively, but not significantly, correlated (*r* = 0.164, *P* = 0.113; Fig. 1C). Age at menarche was significantly associated with variables reflecting longer legs (femoral and tibial lengths) and a relatively narrower body (RBB), as well as larger (midshaft humeral and femoral ToA) and mechanically stronger bones in cross-section (midshaft femoral SSI_p_, midshaft humeral and femoral *I*_max_). Age at menarche was also strongly and significantly negatively associated with absolute and relative subcutaneous fat areas throughout the body (total fat area, fat/muscle area ratios in the upper arms, thigh and calf). Age at menarche was not significantly associated with bone density (TBD). The strongest relationships between age at menarche and bone were again documented in the femur.

The results of regressions investigating the role of developmental (birth weight and age at menarche) and behavioural (sport participation) predictors of lower limb bone size, strength and soft tissue parameters are presented in Table 3 for the upper limb and Table 4 for the lower limb. Developmental factors and physical activity exhibited similar relationships with bone outcomes in the femur; birth weight and age at menarche were both significant predictors of femur length, as well as its ToA and *I*_max_ at midshaft, while sport participation also contributed to femoral ToA, and *I*_max_, as well as SSI_p_ and TBD. By contrast, the dominant influence in the tibia was physical activity, though this influence was largely localised to the midshaft. Sport participation was the clear and sole predictor of ToA, SSI_p_ and *I*_max_ at the highly plastic tibial midshaft, as well as TBD in the distal epiphysis. However, birth weight was the sole significant predictor of bone size in the developmentally canalized variables (tibial length, ToA in the epiphysis). Age at menarche did not significantly predict any tibial bone outcome. In the humeri (left and right values averaged), both sport participation and development (birth weight and/or age at menarche) contributed to predictions of length and midshaft *I*_max_, while sport participation was the sole significant predictor of midshaft ToA and SSI_p_.

**Table 3.** Developmental and behavioural predictors of averaged left and right upper limb outcomes

Outcome	Predictors	*B*	SE	*P*	*r*^2^
Humeral size and cross-sectional parameters					
Humeral length (cm)	Constant	23.8	4.0	<0.001	0.129
	Birth weight (kgs)	0.8	0.4	**0.041**	
	ln(Age at menarche)	2.4	1.5	0.120	
	Sport participation	−0.8	0.4	**0.034**	
ToA: Midshaft (mm^2^)	Constant	110.4	89.6	0.221	0.260
	Birth weight (kgs)	10.7	8.4	0.203	
	ln(Age at menarche)	66.5	33.8	0.053	
	Sport participation	−39.7	8.5	**<0.001**	
ln SSI_p_: Midshaft (mm^3^)	Constant	6.3	0.4	<0.001	0.313
	Birth weight (kgs)	0.05	0.04	0.225	
	ln(Age at menarche)	0.3	0.2	0.120	
	Sport participation	−0.2	0.04	**<0.001**	
ln *I*_max_: Midshaft (mm^4^)	Constant	7.6	0.6	<0.001	0.290
	Birth weight (kgs)	0.1	0.06	0.119	
	ln(Age at menarche)	0.5	0.2	**0.049**	
	Sport participation	−0.3	0.06	**<0.001**	
Upper arm soft tissue parameters (ln)					
Total Muscle Area: Midshaft (mm^2^)	Constant	9.1	0.5	<0.001	0.510
	Birth weight (kgs)	−0.07	0.05	0.169	
	ln(Age at menarche)	−0.2	0.2	0.246	
	Sport participation	−0.4	0.04	**<0.001**	
Total Fat Area: Midshaft (mm^2^)	Constant	11.3	1.1	<0.001	0.242
	Birth weight (kgs)	0.005	0.1	0.965	
	ln(Age at menarche)	−1.5	0.4	**<0.001**	
	Sport participation	0.2	0.1	0.061	
Fat to Muscle Total Area Ratio: Midshaft standardized residuals	Constant	8.1	2.5	0.002	0.257
	Birth weight	0.8	0.3	0.772	
	ln(Age at menarche)	−3.5	0.9	**<0.001**	
	Sport participation	0.5	0.2	**0.037**	

Values from the left and right upper limb were averaged prior to regression analyses; Birth weight was converted to kilograms for regression analyses in order to increase the size of the coefficients for easier interpretation; *P* values in bold indicate significant contribution to predictions at an alpha of <0.05.

**Table 4.** Developmental and behavioural predictors of lower limb outcomes

Outcome	Predictors	*B*	SE	*P*	*r*^2^
Femur size and cross-sectional parameters					
Femur length (cm)	Constant	25.6	5.3	<0.001	0.147
	Birth weight (kgs)	1.1	0.5	**0.031**	
	ln(Age at menarche)	5.7	2.0	**0.006**	
	Sport participation	−0.2	0.5	0.715	
TBD: 4% (mg/cm^3^)	Constant	399.3	56.06	<0.001	0.325
	Birth weight (kgs)	−5.5	5.2	0.291	
	ln(Age at menarche)	−20.0	21.3	0.351	
	Sport participation	−33.6	5.3	**<0.001**	
ToA: 4% (mm^2^)	Constant	1835.3	655.0	0.006	0.242
	Birth weight (kgs)	244.2	60.2	**<0.001**	
	ln(Age at menarche)	342.2	248.6	0.172	
	Sport participation	−159.4	61.9	**0.012**	
ToA: Midshaft (mm^2^)	Constant	275.1	114.6	0.019	0.355
	Birth weight (kgs)	24.0	10.4	**0.024**	
	ln(Age at menarche)	88.7	42.9	**0.041**	
	Sport participation	−61.7	10.9	**<0.001**	
ln SSI_p_: Midshaft (mm^3^)	Constant	7.0	0.3	<0.001	0.304
	Birth weight (kgs)	0.05	0.03	0.132	
	ln(Age at menarche)	0.3	0.1	**0.014**	
	Sport participation	−0.2	0.03	**<0.001**	
ln *I*_max_: Midshaft (mm^4^)	Constant	8.9	0.5	<0.001	0.374
	Birth weight (kgs)	0.1	0.05	**0.020**	
	ln(Age at menarche)	0.5	0.2	**0.026**	
	Sport participation	−0.3	0.05	**<0.001**	
Thigh soft tissue parameters (ln)					
Thigh Total Muscle Area: Midshaft (mm^2^)	Constant	9.8	0.3	<0.001	0.543
	Birth weight (kgs)	0.04	0.03	0.115	
	ln(Age at menarche)	−0.1	0.1	0.467	
	Sport participation	−0.3	0.03	**<0.001**	
Thigh Total Fat Area: Midshaft (mm^2^)	Constant	10.5	0.9	<0.001	0.177
	Birth weight (kgs)	0.1	0.08	0.092	
	ln(Age at menarche)	−1.0	0.3	**0.006**	
	Sport participation	0.2	0.1	**0.014**	
Thigh Fat to Muscle Total Area Ratio: Midshaft standardized residuals	Constant	4.3	2.3	0.065	0.212
	Birth weight	0.3	0.2	0.124	
	ln(Age at menarche)	−2.5	0.9	**0.005**	
	Sport participation	0.7	0.2	**0.004**	
Tibial size and cross-sectional parameters					
Tibial length (cms)	Constant	24.6	5.6	<0.001	0.144
	Birth weight (kgs)	1.3	0.5	**0.012**	
	ln(Age at menarche)	3.6	2.1	0.095	
	Sport participation	−1.0	0.4	0.066	
TBD: 4% (mg/cm^3^)	Constant	504.7	74.9	<0.001	0.226
	Birth weight (kg)	−12.0	6.9	0.086	
	ln(Age at menarche)	−47.1	28.4	0.100	
	Sport participation	−32.1	7.1	**<0.001**	
ToA: 4% (mm^2^)	Constant	499.1	276.0	0.074	0.244
	Birth weight (kgs)	100.2	25.4	**<0.001**	
	ln(Age at menarche)	139.9	104.5	0.184	
	Sport participation	−76.6	26.1	**0.004**	
ToA: Midshaft (mm^2^)	Constant	311.6	109.9	0.006	0.272
	Birth weight (kgs)	19.0	10.1	0.064	
	ln(Age at menarche)	41.8	41.6	0.318	
	Sport participation	−53.9	10.4	**<0.001**	
ln SSI_p_: Midshaft (mm^3^)	Constant	7.4	0.4	<0.001	0.227
	Birth weight (kgs)	0.06	0.04	0.107	
	ln(Age at menarche)	0.03	0.2	0.824	
	Sport participation	−0.2	0.04	**<0.001**	
ln *I*_max_: Midshaft (mm^4^)	Constant	9.5	0.6	<0.001	0.321
	Birth weight (kgs)	0.08	0.05	0.128	
	ln(Age at menarche)	0.2	0.2	0.293	
	Sport participation	−0.3	0.1	**<0.001**	
Calf soft tissue parameters (ln)					
Calf Total Muscle Area: Midshaft (mm^2^)	Constant	9.2	0.3	<0.001	0.138
	Birth weight (kgs)	0.03	0.03	0.396	
	ln(Age at menarche)	−0.1	0.1	0.268	
	Sport participation	−0.1	0.03	**0.001**	
Calf Total Fat Area: Midshaft (mm^2^)	Constant	9.9	0.8	<0.001	0.224
	Birth weight (kgs)	0.1	0.07	0.053	
	ln(Age at menarche)	−1.1	0.3	**<0.001**	
	Sport participation	0.2	0.1	**0.011**	
Calf Fat to Muscle Total Area: Midshaft standardized residuals	Constant	5.4	2.0	0.009	0.246
	Birth weight	0.4	0.2	0.061	
	ln(Age at menarche)	−2.9	0.8	**<0.001**	
	Sport participation	0.6	0.2	**0.002**	

Birth weight was converted to kilograms for regression analyses in order to increase the size of the coefficients for easier interpretation; *P* values in bold indicate significant contribution to predictions at an alpha of <0.05.

In all limb segments analysed, age at menarche was most strongly associated with adiposity. Age at menarche was significantly negatively associated with total fat areas and fat/muscle area ratios across all upper and lower limb sites, and was also a significant predictor of total fat area and fat to muscle area in the upper arms, thigh and calf. Age at menarche was the sole significant predictor of mean adiposity in both upper limbs, and was a stronger predictor of thigh and calf adiposity than was sport participation. In contrast, sport participation was the sole predictor of total muscle area at all sites examined.

## DISCUSSION

The current study documents the associations of life history traits reflecting maternal investment and maturational rate with indices of body composition and the mechanical competence of the adult female skeleton. We found that variation in female life history strategy, specifically relative investment during development in growth and maintenance versus reproduction, is associated with bone mechanical competence in adulthood, extending the influence of both maternal investment and maturational rate to bone fracture risk.

Our results show that higher maternal investment contributes to greater growth in the next generation, a marker of a slower life history trajectory [9], as indicated by larger body size, leanness and absolute mechanical competence of the skeleton. In contrast, lower maternal investment is associated with markers of a faster life history trajectory—smaller adult size, indicative of earlier growth cessation and reduced investment in skeletal size and mechanical competence. The association of birth weight with age at menarche was in the expected direction, with larger birth weight predicting slower maturation, but the correlation did not achieve significance (*P* = 0.113). One contributing factor may have been the lack of availability of gestational age, preventing us from evaluating fetal weight gain in terms of the time available for maternal investment.

Our other marker of a faster life history trajectory, earlier maturation, was likewise associated with the diversion of resources towards adiposity and the earlier attainment of sexual maturity, suggesting greater investment in short-term survival and reproduction. It is likely that the relationship between earlier menarche and greater adult adiposity documented here indirectly reflects the relationship between *childhood* adiposity and age at menarche, as adult adiposity is more strongly associated with the former than the latter [28]. Relatively higher fat in childhood, *prior* to the onset of puberty, is associated with an earlier age at menarche [28, 29], and childhood adiposity itself is heavily influenced by growth patterns much earlier in life. Specifically, rapid infant weight gain, particularly in the first 9 months to 1 year of life is associated with earlier age at menarche [30–33] and an elevated risk of childhood obesity (as reflected by higher childhood weight, BMI and fat index) that extends into adulthood [31, 33, 34].

As posited by Wells [9], the pace of early life growth may be significantly impacted by variation in maternal life history strategy, and the extent to which the mother partitions nutritional investment between pregnancy and lactation. Mothers with fast life history trajectories, who invested more in reproduction during their own development (indicated by earlier menarche, smaller adult size and greater adiposity) have somatic traits that constrain fetal growth and reduce offspring birth weight. These mothers may compensate for this by a greater reliance on nutritional investment via lactation, made possible by the greater accumulation of adiposity earlier in their development. Indeed, both earlier maternal age at menarche [33] and low infant birth weight relative to length [32, 35] are associated with rapid infant growth and accelerated pubertal maturation.

Of relevance to our primary outcome, the faster developmental trajectory associated with these maternal factors not only results in a smaller body with more adipose tissue, but it also builds a mechanically weaker skeleton in absolute terms. We therefore provide empirical data supporting the hypothesis that life history trajectory shapes components of health associated with ‘maintenance’ [9], namely that greater maternal investment during fetal life (proxied by larger birth weight) and later menarche each prioritize the attainment of larger adult body size and somatic quality.

### Why is a larger bone more mechanically competent?

Higher birth weight and later age at menarche are both associated with longer limb bones that are wider in cross-section at the epiphyses and/or at the midshaft. This arrangement has biomechanical implications for bone strength: bending and torsion are the most mechanically relevant strains acting on the lower limb bones during locomotion, and these strains increase proportionately outwards as distance from the neutral centroid or bending axis of the cross-section increases [23, 26]. As a result, bone that is located farther from the neutral centroid or bending axis is subjected to the most strain during loading and this bone is very important for maximizing bending/torsional rigidity. A large bone has a greater distance between the cortical bone tissue and the centroid/bending axis in cross-section than a smaller bone, so it has higher bending/torsional rigidity simply as a bi-product of size. This is why statistical analyses controlling for body size variation are essential when comparing bone strength between individuals of differing body size [23, 36]. Because higher birth weight, a later age at menarche, and a slower life history trajectory contribute to a larger body and larger bones, they also indirectly contribute to a mechanically stronger limb bone in absolute terms.

### The combined influence of development and behaviour on the female skeleton

Various environmental factors, such as mechanical loading, can alter the basic size-strength relationship of a limb bone shaft, such that a small bone can become *relatively* strong, by either adding new bone tissue, or redistributing existing bone, as far as possible from the neutral bending axis or centroid [18, 19, 23, 37–42]. Theoretically, this functional adaptation of bone in response to mechanical loading should be highest in regions of the skeleton where variation in geometry is least developmentally or functionally constrained, for example in limb bone midshafts relative to external epiphyseal dimensions or bone lengths. These patterns of plasticity to mechanical loading within bones are consistently identified in experimental and anthropological research [e.g. 14–17]. Previous studies have documented particularly high plasticity to mechanical loading at the tibial midshaft, a region subject to fewer body mass and breadth-related functional constraints than the femoral midshaft and fewer locomotor and articular constraints than its more distal locations [47–50].

Variation in skeletal plasticity in response to developmental and environmental factors is evident in the results of the current study as well. Even when controlling for athletic participation, the high functional plasticity of the tibial midshaft in this group of women, comprised in large part of competitive athletes, is evident: there are few significant relationships between developmental parameters and bone outcomes at the tibial midshaft, especially relative to the bone’s more highly canalized parameters (e.g. maximum length and external joint size), and relative to the femoral midshaft. Similarly, in regression analyses, birth weight is a significant predictor of developmentally canalized variables like tibial length and distal tibial epiphyseal bone area, but the influence of physical activity vastly outweighs that of developmental parameters at the tibial midshaft, where sport participation is the sole significant predictor of midshaft size and mechanical competence. This highlights the role of development in shaping the absolute size and strength of the tibia, and the essential contribution of subsequent physical activity in then increasing relative strength to meet mechanical demands.

A similar pattern is documented in the humerus, despite the non-weight-bearing environment of the upper limb. When incorporating the influence of sport participation with that of developmental factors on humeral outcomes, birth weight is a significant predictor of bone length, but the influence of physical activity again vastly outweighs that of developmental parameters at midshaft. The similarity in the relationships between physical activity, development and bone outcomes in the humerus and tibia, despite the non-weight-bearing environment of the former, may be due in part to the relatively high upper limb loading of the women included in the current study. Though control subjects and soccer players (combined *N* = 49) reported very little history of upper limb loading, past involvement was reported in the following activities among the other athletes recruited (*N* = 62), involving at least 4+ h a week for ≥ 1 year and often at a competitive level: weight-training, rowing, field and ice hockey, rugby, netball, boxing, kayaking, cricket, rounders, tennis, gymnastics, lacrosse, volleyball, karate/martial arts and cheerleading/tumbling. As a result, humeral morphology among many of the women in the study group is likely reflecting substantial adaptation to mechanical loading, so relative differences in loading between the upper and lower limbs may be minimized. However, when controlling for physical activity in partial correlation analyses, the influence of development on the morphology of the humerus did occasionally differ from that of the weight-bearing lower limb bones. For example, birth weight was significantly associated with ToA in the femur and tibia but not the humeri, which may be reflecting an influence of body size and shape on lower limb bone cross-sectional size.

In the current study, it is femoral bone outcomes that consistently demonstrate the strongest correlations with birth weight and age at menarche, and it is the only bone analysed in which developmental parameters retain significant predictive capacity for midshaft morphology even when physical activity is considered. These results support previous work suggesting that femoral morphology is subject to the influence of body size and shape to a greater extent than the humerus or tibia. Femoral morphology, even at midshaft, is thought to be influenced by locomotory, climatic and obstetric selection pressures acting on the body size and/or the relative breadth of the pelvis [48, 50–53]. Our results suggest that developmental influences on body size and breadth may also be reflected in the greater predictive capacity of birth weight and age at menarche on midshaft geometry in the femur relative to the humerus and tibia.

Developmental and behavioural parameters also interact in an interesting way to govern adult soft tissue. Muscle tissue is strongly influenced by sport participation in our analysis, whereas adiposity reflects a more complex set of influences that include both behaviour and maturational rate, which itself may indirectly reflect factors not considered here, such as infant growth patterns. Our results demonstrate that a life history trajectory that prioritizes short-term survival/reproduction over long-term growth/maintenance does so through strong investment in adiposity at the expense of body size, bone size and bone strength. The relationship between earlier age at menarche and investment in adiposity is so strong that age at menarche is the sole significant predictor of adult adiposity in the upper limb and, in the lower limb, remains a stronger predictor of adiposity than intensive sport participation.

Interestingly, total bone density bears no relationship with developmental variables at any of the lower limb bones examined, and it is sport participation that is the sole predictor of TBD in the lower limb sites in regression analyses; this relationship between bone density and mechanical loading is well-established in the literature [41, 54, 55]. The lack of relationship between TBD and developmental parameters likely reflects the independence of bone density and size; because developmental parameters act on mechanical strength largely through their influence on bone size, variation within them should have no bearing on a size-independent parameter. Further, bone density is influenced by a variety of factors in addition to physical activity that were not considered in this study, including genes [16], diet [56], hormonal contraceptive use [57–59] and menstrual history [60, 61].

Though bone density and its age-related loss are important etiological factors in osteopenia/osteoporosis risk, these diseases are complex; low bone mineral status does not necessarily equate to high osteoporosis or fracture risk [62]. Thus, though maternal investment and maturational rate do not appear to be important determinants of bone density in lower limb bone epiphyses, they contribute to limb bone mechanical strength through their impact on absolute bone size and its spatial distribution, contributions that themselves may have important implications for fracture risk.

## LIMITATIONS

One limitation of the study was the lack of information on gestational age (number of weeks gestation). The extent to which birth weight is indicative of maternal investment during foetal life would be improved if the duration of gestation could have been taken into account. Birth weight for gestational age provides a marker of the rate of foetal growth, and is strongly predictive of cardiovascular health later in life [63]. Both gestational age and birth weight were significant predictors of age at menarche in our earlier study of South Asian women [13], indicating that higher maternal investment was associated with a slower maturational rate. By not accounting for gestational age, we may be missing an important component of maternal investment, that of foetal growth rate, which may explain the non-significant correlations between birth weight and age at menarche in this study, in contrast to our previous findings [13].

## CONCLUSIONS AND IMPLICATIONS

Though physical activity is one of the main determinants of adult female bone strength, the significant contributions of maternal investment and maturational rate to bone mechanical competence, independent of activity level, extend the influence of life history trajectory to size-dependent markers of fracture risk as well. Consistent with a broader model [9], a slower life history trajectory is associated with greater investment in ‘maintenance’, which enhances skeletal size and mechanical integrity in addition to reducing the longer-term risk of non-communicable disease. Physical activity during the lifespan appears to then impact this relationship between life history and absolute skeletal size-strength relationships, by increasing the relative strength of bone when mechanical loading is sufficient to require it, but predominantly in the less developmentally canalized regions of the bone, such as the midshaft region, and in bones less influenced by body size variation, such as the tibia and humerus. Physical activity was also the sole predictor of total bone density in the joints of the lower limb, demonstrating its own important contribution to osteoporosis and fracture risk. The improvement of maternal health during pregnancy and the encouragement of physical activity in girls and women are thus both important factors that together might help increase bone quality and mechanical competence in the female skeleton and reduce the risk of future fracture.

## DECLARATIONS OF FUNDING

The research leading to these results has received funding from the European Research Council under the European Union’s Seventh Framework Programme (FP/2007-2013)/ERC Grant Agreement n.617627 (J.T.S.).
